# LigninFit: Stochastic Simulation Software for the Prediction and Analysis of Experimentally Observed Lignin Molecules

**DOI:** 10.34133/csbj.0062

**Published:** 2026-09-07

**Authors:** Lianne Gahan, Adélaïde Raguin, Partho Sakha De

**Affiliations:** ^1^ Institute for Computational Cell Biology, Heinrich Heine University, 40225 Düsseldorf, NRW, Germany.; ^2^ Cluster of Excellence on Plant Science (CEPLAS), Heinrich Heine University, 40225 Düsseldorf, NRW, Germany.; ^3^ Bioeconomy Science Center (BioSC), c/o Forschungszentrum Jülich, 52425 Jülich, NRW, Germany.; ^4^ Laboratoire Bordelais de Recherche en Informatique (LaBRI - UMR 5800), CNRS, Univ. Bordeaux, Bordeaux INP, 33405 Talence, France.

## Abstract

Lignin’s complex and highly branched architecture plays a critical role in biomass utilisation. Yet, unravelling the intricate relation between lignin’s measurable structural features and the underlying biosynthesis dynamics remains a major challenge. This difficulty stems from the variability in monolignol and bond type compositions, experimental datasets with typically distinct degrees of completeness, and extraction methods that differently impact molecular features. Together, this highlights the need for advanced numerical frameworks able to rationalise experimental data. We introduce LigninFit, a simulation software that integrates Lignin-KMC within an efficient and modular parameter optimisation procedure to infer lignin biosynthesis dynamics from bond distributions. Using experimental data from a curated set of 20 biomass samples, we reproduce their bond distributions following 3 versions of the fitting procedure, differing in the number of model parameters optimised. We then generate extensive *in silico* libraries of lignin structures for the best fit of each biomass and analysein depth, at both the ensemble and molecular levels, the properties of the resulting molecules in terms of bond distributions and branching degrees. Overall, LigninFit not only allows for predicting the complete bond distribution of incomplete datasets, and highlighting unexpected similarities and differences across biomass types, but also reveals which parameters most strongly impact the biosynthesis dynamics and which metrics are most suitable for discriminating among biomass types. Eventually, as a modular and open-source software, LigninFit paves the way for additional modelling developments, thereby contributing to guide experimental endeavours.

## Graphical Abstract

**Figure:**
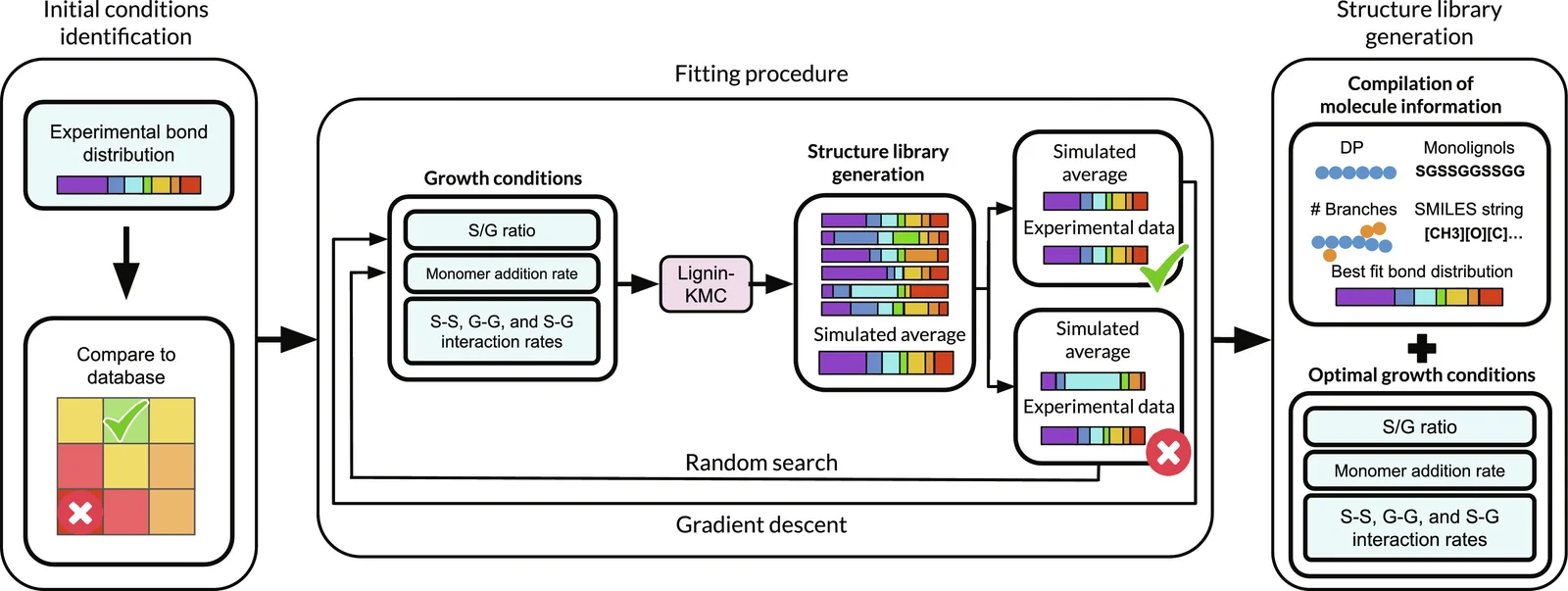

## Introduction

Plant cells are surrounded by complex and composite polymer-based cell walls that can be classified into the primary cell wall made of cellulose, hemicellulose, and pectin only, and the secondary cell wall that additionally contains lignin [1]. Lignin, which makes ca. 15% to 30% of biomass [2], provides crucial structural support to plants by enhancing the rigidity and strength of cell walls, particularly in vascular tissues such as xylem and phloem [3,4]. This rigidity is essential for plants to maintain an upright posture and grow tall, making it advantageous to capture sunlight and maximise photosynthesis. The presence of lignin in the secondary cell walls of plant cells [1,5,6] such as fibres and tracheids, ensures that these cells can withstand various mechanical stresses, including wind and physical damage [7]. Lignin also plays an important role in the efficient transport of water within plants, which allows for nutrient transport and overall plant health [1,6]. By forming a hydrophobic barrier within the xylem vessels, it prevents water loss and facilitates the uninterrupted flow of water from the roots to the leaves. This hydrophobic nature is critical in maintaining the cohesion–tension mechanism that drives water transport [8]. Additionally, the structural rigidity provided by lignin ensures that xylem vessels do not collapse under the negative pressure generated during transpiration [9].

Lignin is a large, complex, and highly aromatic macromolecule made of building blocks called monolignols, of which the main 3 are p-coumaryl alcohol (H), coniferyl alcohol (G), and sinapyl alcohol (S) [10]. Different biomasses can typically be characterised by their distinct monolignol compositions [11]. For example, softwoods primarily contain G units, while hardwoods show a combination of both S and G monolignols. Although H monolignols are detected in low quantities in both hardwoods and softwoods, it is more common in grass species. The bonds that form between the lignin subunits are complex and varied [3,12]. The 7 typically observed ones are a combination of carbon–oxygen (*β*–*O*–4, α–*O*–4, and 4–*O*–5) and carbon–carbon linkages (*β*–*β*, *β*–5, *β*–1, and 5–5) [13], which may also involve the formation of branches, giving rise to lignin molecules with highly complex structures. The biosynthesis of lignin takes place in 2 steps. First, the monolignol precursors are synthesised in the cytosol and transported to the apoplast through the membrane. Second, their activated form, possibly aided by oxidative enzymes such as laccases and peroxidases, undergoes stochastic radical polymerisation, whose kinetic rates are not only governed by the respective chemical probabilities of bond formation but also spatially constrained and biologically regulated by the plant’s biology, giving rise to the lignin molecule [11,14–16]. However, despite several decades of research, the anabolic biosynthesis of lignin remains largely unclear [3,17].

Experimentally, it is possible to determine monolignol composition as well as the ratio of the respective abundances for S and G monolignols (noted as the S/G ratio). However, structural characterisation is typically performed on samples comprising a large number of molecules that are highly heterogeneous and dispersed. Thus, the details of the lignin features are only accessible as an average of complex distributions and not at the single-molecule level. In addition, as highlighted above, lignin molecules are highly complex, and they vary not only depending on the plant considered but also for different tissues of a given species. Eventually, although lignin biosynthesis is a well-known process, its full and detailed understanding remains elusive. Besides, lignin, as a key player in the water transport and mechanical properties of the plant cell wall, is of primary interest for application domains relating to breeding drought-resistant new crops in the context of climate change and biotechnologies for biofuel production in the context of energy shortage [18]. Hence, despite a clear agronomic and bioeconomic value, our fundamental understanding of lignin remains extremely limited, and both its molecular structure and growth process are so far poorly understood.

To support experimental developments, computational modelling approaches have been implemented. Focusing on those that are open source, we note that Eswaran et al. [19] took a “top-down” approach, wherein lignin molecules are generated based upon an input bond distribution. While their lignin structure generator (LGS) replicates the input bond distributions and monolignol compositions for spruce and birch samples, it does so when being limited to molecules with degree of polymerisation (DP) ≤ 25 and without considering the free-energy barriers specific to the formation of each bond type. Precisely, the polymerisation dynamics is not modelled as such, and instead molecules are generated as combinations of fixed input bond distributions and S/G ratios, which does not capture the physics and biochemistry of the process. Besides, LigninGraphs [20] is a visualisation software that offers to view multi-scale details of molecules produced by the aggregation of monomers with fixed monolignol and bond compositions, which are fed from experimental data. Eventually, Orella et al. [21] built the Lignin-KMC toolkit, a “bottom-up” approach based on a kinetic Monte Carlo algorithm to generate lignin molecules from initial monolignol composition. The model produces a library of diverse molecules, from which the authors made a limited comparison to experimental data, for poplar only. Thus, the study neither focuses on finding the growth conditions that allow quantitatively reproducing specific experimental data nor considers various biomasses. Additionally, analysing simulation outcomes at the single-molecule level with Lignin-KMC remains highly challenging.

As a consequence, so far, despite the few modelling approaches developed, a systematic comparison between simulations and experimental findings for more than a few illustrative plant sources and the possibility to analyse single-molecule information are clearly lacking, which strongly hinders the transferability of modelling results to support rationalising experimental findings. In this study, we specifically focus on this 2-fold challenge. To bridge this gap, we set out by collecting a substantial library of experimental data for 20 different biomasses. Then, we develop a complex computational framework, LigninFit, which at its core encapsulates Lignin-KMC into a fitting procedure to allow for computationally efficient and quantitative comparison of the simulation outcomes with experimental data of each biomass sample. Then, we examine the bond distributions and S/G ratios of *in silico* generated lignin libraries and analyse the growth properties that result in these distributions. We also demonstrate how our method predicts the remaining parts of a partial bond distribution, which typically consists of rarer bond types that are challenging to measure accurately through experimental methods. Then, we discuss the main physical properties of the *in silico* generated lignin structures at the molecular level. Lastly, we conduct a systematic analysis of their spatial branching properties using different metrics and compare the differences across biomass types.

## Methods

### Experimental data collection

Table 1 shows the available bond distributions, monolignol abundances, and physical features for 20 different biomasses, which are categorised based on the type of plant source, i.e., Hi for hardwoods, Si for softwoods, Gi for grasses, and Xi for others. We include findings from different studies for the same biomass (e.g., for birch, beech, and spruce woods) to highlight the variability in the data. The relative bond abundances were experimentally quantified from the analysis of nuclear magnetic resonance (NMR) spectra, the S/G ratio and the individual monolignol relative abundances from gas chromatography/mass spectrometry (GC/MS) spectra analysis, and the *M_w_* (weight average molecular weight) and *M_n_* (number average molecular weight) values from gel permeation chromatography, while the mean DP was calculated from the S/G ratios and the *M_w_* and *M_n_* values and the molecular weight of the individual monolignol units. Columns H2, H3, and H4 indicate differences of up to 32% for the proportions of S and G monolignols, for beech. Similarly, columns H5 [19] and H6 [22] indicate that the *β*–*O*–4 percentage alone can vary by 9% and the *β*–*β* percentage by 15%, for birch. Since the simulation software Lignin-KMC only considers S- and G-type monolignols, we focus on biomasses with relatively low fractions of H monolignols (or other types). In many cases, the experimental data available are only for the most abundant bond types, e.g., *β*–*O*–4, *β*–5, and *β*–*β*, owing to the challenges of collecting a complete bond distribution in experiments.

**Table 1.** Experimentally determined bond distributions, monolignol abundances (including S/G ratios), and physical features (including degrees of polymerisation, weight average $M_{w}$, and number average $M_{n}$). The “*” next to a bond distribution value indicates that the value represents the sum of 4–*O*–5, 5–5, and α–O–4 bond types. The alphabetical entries in the rows titled “Reference” correspond to the following references as the source of experimental data: a [35], b [46], c [47], d [22], e [37], f [48], g [49], h [19], i [20], j [50], k [51], l [38], m [52], n [53], and o [54].

Tag	H1	H2	H3	H4	H5	H6	H7	S1	S2	S3	S4	S5	G1	G2	G3	G4	X1	X2	X3	X4
Biomass	Eucalyptus	Beech	Beech	Beech	Birch	Birch	Poplar	Spruce	Spruce	Black spruce	Norway spruce	Pine	Miscanthus	Miscanthus	Wheat straw	Wheat straw	Sida	Rapeseed	Silphium	Pineapple
Bond distributions																				
β–O–4	0.69	0.60	0.60	0.84	0.71	0.62	0.78	0.51	0.67	0.49	0.45	0.66	0.68	0.68	0.77	0.77	0.86	0.88	0.84	0.95
β–β	0.18	0.10	0.08	0.16	0.18	0.03	0.15	0.02	0.06	0.06	0.03	0.16	0.17	0.17	0.04	0.04	0.13	0.08	0.13	0.01
β–5	0.03	0.06	0.01	0.03	0.06	0.06	0.07	0.12	0.11	0.13	0.11	0.18	0.15	0.15	0.11	0.11	0.04	0.05	0.04	0.05
β–1	0.03	0.15	0.03	-	0.00	0.07	-	0.10	0.00	0.01	0.01	-	0.00	0.00	0.03	0.03	-	-	-	-
5–5	0.07*	0.02	0.28*	-	0.00	0.07	0.00	0.13	0.06	0.31*	0.40*	0.00	0.00	0.00*	0.03	0.05*	-	-	-	-
4–O–5	-	0.02	-	-	0.06	0.07	-	0.04	0.06	-	-	-	0.00	-	0.00	-	-	-	-	-
α–O–4	-	0.05	-	-	0.00	0.08	-	0.08	0.06	-	-	-	0.00	-	0.02	-	-	-	-	-
Reference	a,b	d	a,e	f	g,h	d	i	d	h,k,l	a,e	a,e	i	d,m	a,m	d	a,o	f	f	f	f
Monolignol abundances																				
S/G ratio	5.53	0.56	0.56	2.13	1.00	1.00	1.70	0.01	0.00	0.00	0.00	0.00	1.09	0.85	0.47	0.47	1.06	0.71	1.18	0.10
S	0.83	0.36	0.36	0.68	0.50	0.50	0.63	0.01	0.00	0.00	0.00	0.00	0.50	0.44	0.30	0.30	0.51	0.41	0.49	0.08
G H	0.15	0.64	0.64	0.32	0.50	0.50	0.37	0.94	1.00	0.98	0.98	0.98	0.46	0.52	0.64	0.64	0.48	0.58	0.42	0.83
0.02	0.00	0.00	0.00	0.00	0.00	0.00	0.05	0.00	0.02	0.02	0.02	0.04	0.04	0.06	0.06	0.03	0.02	0.10	0.09
Reference	a,c	a,d	a,d	f	g,h	d	i	d	h,k,l	a,e	a,e	i	d,m	a,m	d	a,o	f	f	f	f
Physical features																				
DP	14.86	26.24	26.24	23.13	12.68	12.68	8.39	48.81	-	-	-	37.10	7.96	8.16	12.55	12.48	-	-	-	-
*M_w_*	6700	5510	5510	5510	4600	4600	3550	23500	3700	-	-	14900	2310	13700	4210	4210	-	-	-	-
*M_n_*	2600	3690	3690	3690	1880	1880	1310	6400	-	-	-	4700	1240	1240	1850	1840	-	-	-	-
Reference	a,c	a,d	a,d	a,d	a,d	a,d	a,j	d	h,k,l	-	-	a	a,m	a,m	a,n	a,n	-	-	-	-

### Computational framework

**Fig. 1.**
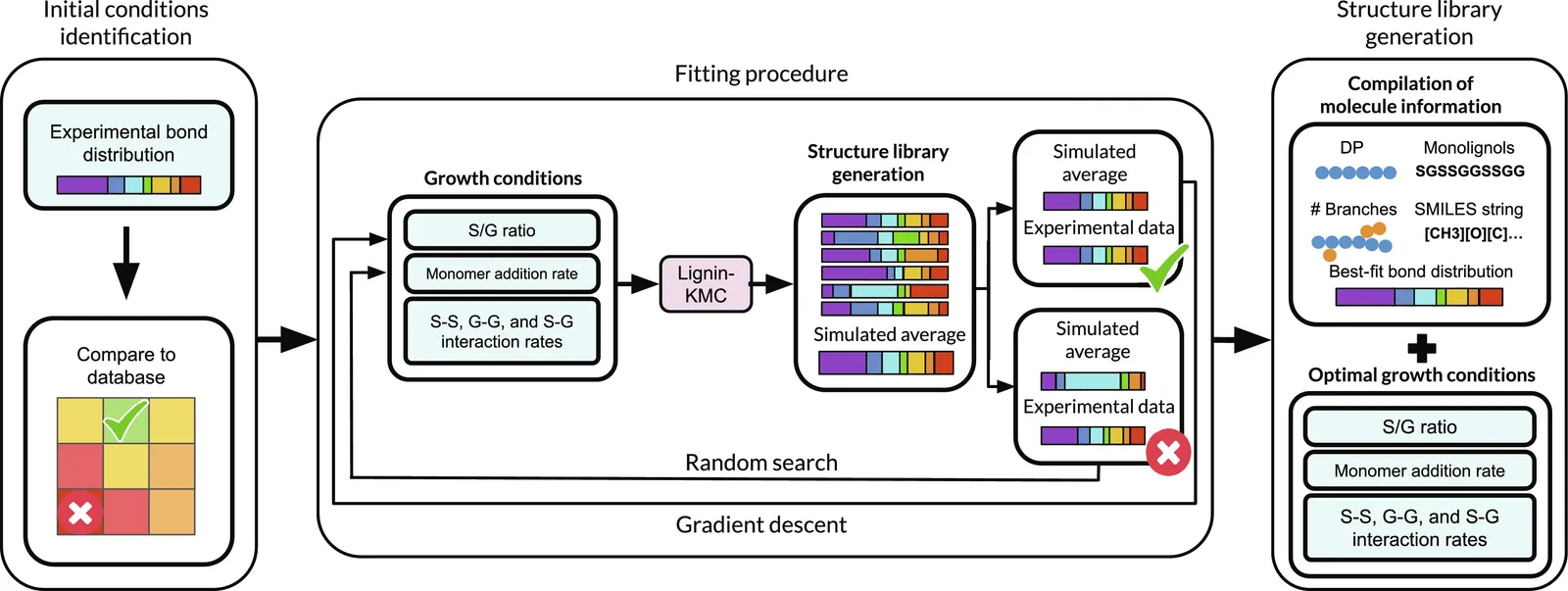
Scheme of the computational framework LigninFit. In the leftmost box, the “Initial conditions identification” finds the starting parameters for the “Fitting procedure” by comparing the target experimental bond distribution to an existing simulated database of bond distributions built by caching results of previous runs, following a grid search. In a loop, the “Fitting procedure” optimises the input parameters (S/G ratio, monomer addition rate, and the free-energy barriers of S–S, G–G, and S–G bond formation) such that the simulated bond distribution matches the target experimental one. Following the identification of the best fit, a large library of molecules is generated for the corresponding set of parameter values, leading to the “Compilation of molecule information” and the identification of the “Optimal growth conditions” (rightmost box).

LigninFit is the overall computational framework that we built in Python to perform a systematic and quantitative comparison of experimental data with simulated lignin molecules. It makes use of the previously published Lignin-KMC simulation algorithm [21], and its detailed architecture is illustrated in Fig. 1 and explained in further details in the following subsections.

#### Initial conditions identification

**Figure B.1:**
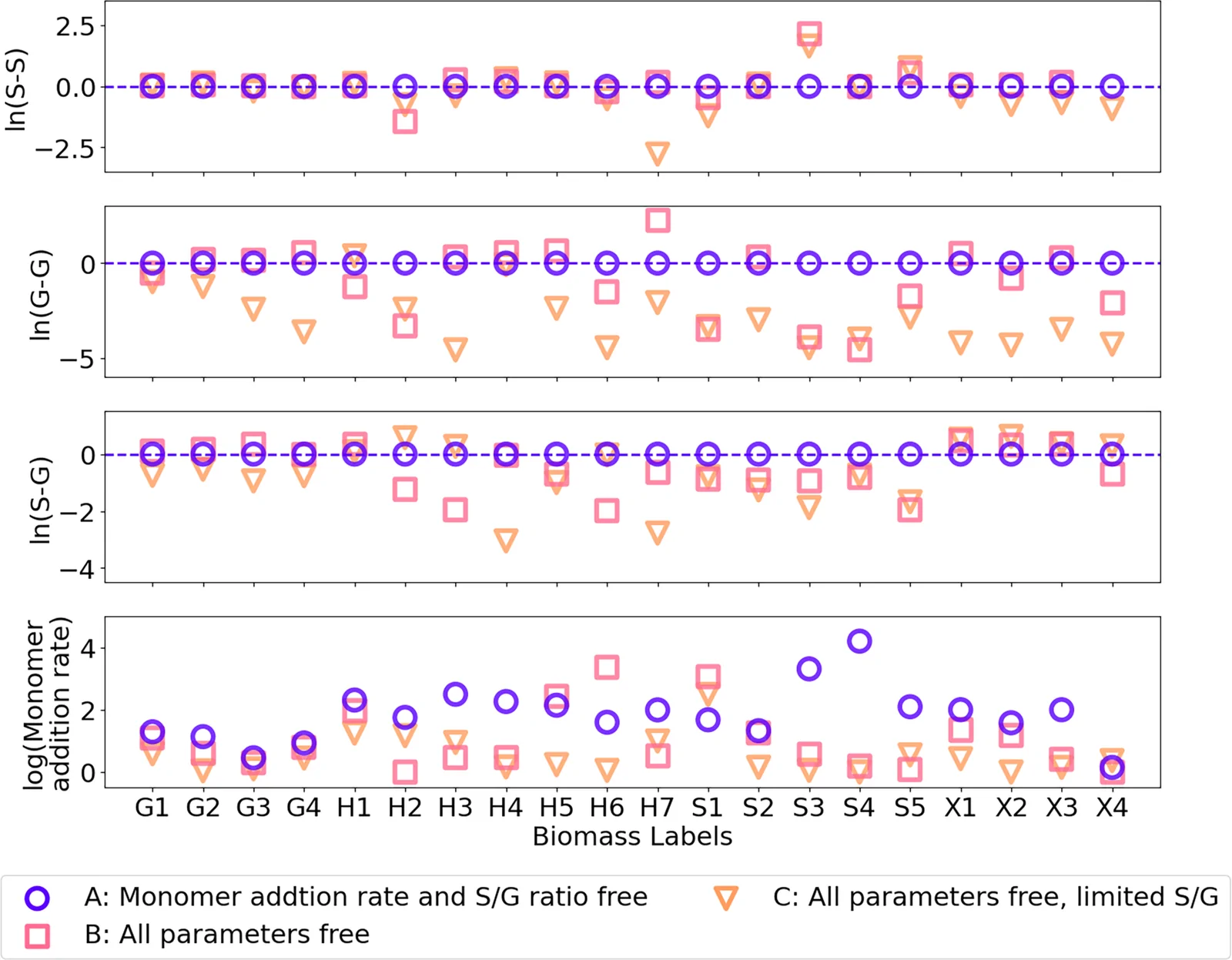
Best-fit parameter values for reproducing the experimentally measured bond distributions shown in Fig. 3i: (top 3 rows) scaling parameters for the free-energy barriers of the formation of S–S, G–G, and S–G bonds; (bottom row) the corresponding monomer addition rate values. The colour-coding for sets A, B, and C is similar to that of Fig. 3ii.

As shown in the leftmost box of Fig. 1, LigninFit includes an initial automated search of our compiled library of lignin structures to identify the preexisting ones that best capture the target experimental bond distribution data, thereby determining the best starting parameter set for the Fitting procedure (shown in the middle box). To perform this initial search, 3 options are possible:

1. No search: No search is performed, and the initial conditions for the Fitting procedure (in the middle box) are manually entered.

2. Database search: Ranges are provided for the parameters that are chosen to be varied. These can be the S/G ratio (i.e., the ratio of S to G monolignols that are added to the simulation system), the monomer addition rate (i.e., the rate at which monomers are added to the simulation system), the S–S, G–G, and S–G scaling parameters (i.e., the scaling parameters for the free-energy barriers of bond formation, which are converted into interaction rates within Lignin-KMC), and the minimum and maximum number of monomers (i.e., the number of monolignols in the system when the simulation starts and the maximum it can contain, respectively). In the sets referred as A, B, and C in Fig. 3 and Fig. B.1, and in Table 3, the parameters varied for each set are as follows: A—S/G ratio and monomer addition rate; B—S/G ratio, monomer addition rate, and the scaling parameters for the free-energy barriers of S–S, G–G, and S–G bond formation; C—monomer addition rate, the scaling parameters for the free-energy barriers of S–S, G–G, and S–G bond formation, while the S/G ratio is allowed to vary between ±0.5 from the experimentally measured value (or in the range 0 to 1 if the experimentally measured value is less than 0.5). In all the sets A, B, and C, the minimum number of monomers is set to 10 and the maximum number of monomers to 500. The bond distribution of the target experimental biomass is compared to preexisting simulation results stored in our database, which is consistently incremented using the results of earlier simulation runs and their input parameter sets. Within this database, the closest matching bond distribution (with the lowest Euclidean distance) that respects the value ranges provided for each parameter is identified. This step is designed to reduce repeat simulations and save computation time.

3. Simulations search: Ranges are provided for the *N* parameters that are chosen to be varied, together with the number of different values (noted *M*) they each can take. Then, during this initial search step, Lignin-KMC simulations are performed for these $N^{M}$ sets of parameter values, and the closest matching bond distribution (with the lowest Euclidean distance) that respects the range of values provided for each parameter is identified.

#### A brief reminder of Lignin-KMC

**Fig. 2.**
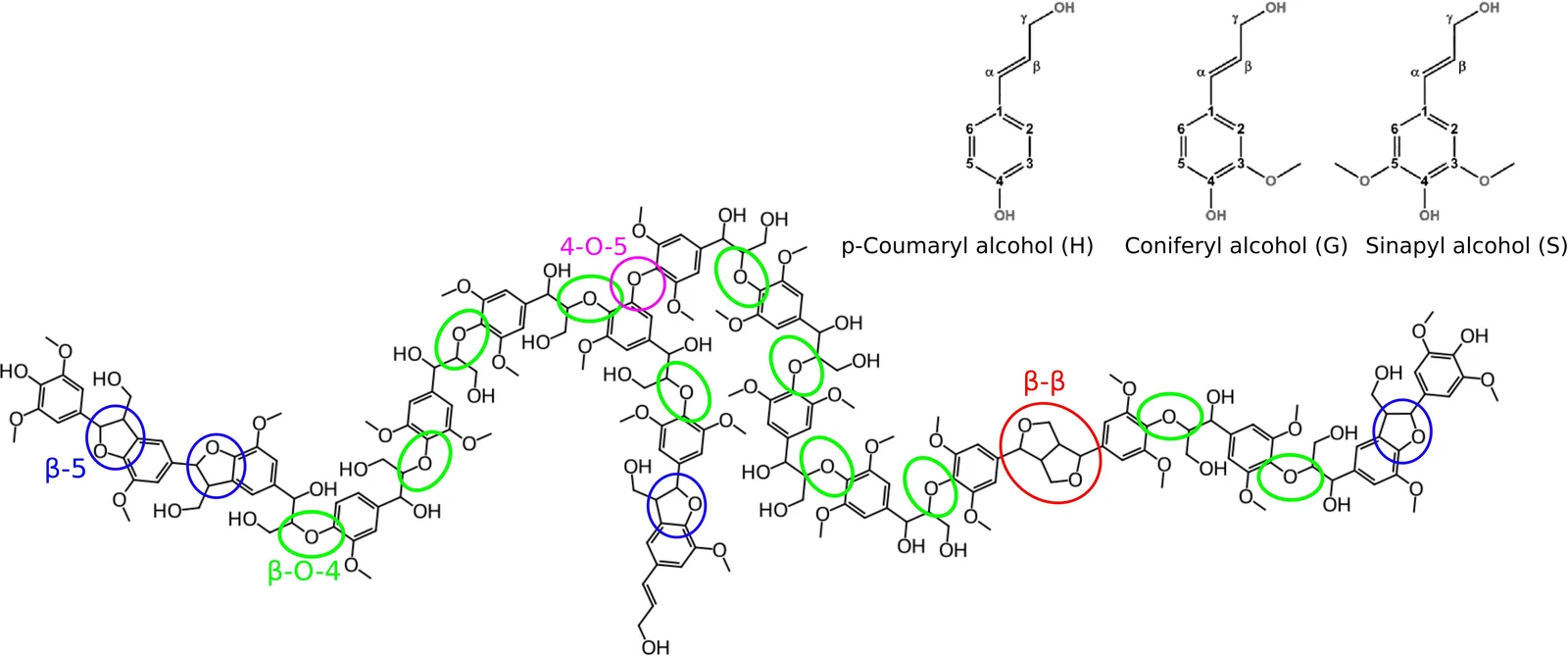
RDKit-based visualisation [45] of the molecular structure of a birch molecule with degree of polymerisation DP = 18 generated by LigninFit. The structure contains 11 *β*–*O*–4 linkages (green), 4 *β*–5 linkages (blue), 1 *β*–*β* linkage (red), and 1 4–*O*–5 linkage (magenta). The structure displays carbon atoms in the cyclic backbone. Hydrogen atoms are omitted for clarity. (Inset) H, G, and S monolignols, with carbon atoms marked.

Lignin-KMC has been developed by Orella et al. [21] as an open-source Python software that allows generating libraries of lignin molecules, according to an aggregation process that follows a kinetic Monte Carlo algorithm. It accounts for the formation of 7 bond types, i.e., *β*–O–4, *β*–*β*, *β*–5, 5–5, α–*O*–4, 4–*O*–*5*, and *β*–1, whose binding rates are monolignol specific and derived from density functional theory calculations [23]. Lignin-KMC relies on varying the 4 following parameters: the monomer addition rate, the minimum and maximum monomer numbers, and the S/G ratio, unlike the energy barriers for bond formation. Besides generating the library of molecules, Lignin-KMC provides the total number of molecules generated in that library, the average bond distribution and degree of polymerisation, the branches and bonds formed, and the full SMILES (simplified molecular input line entry system) strings for each of the molecules produced, which can be turned into 2-dimensional representations, like shown in Fig. 2.

#### Fitting procedure

**Fig. 3.**
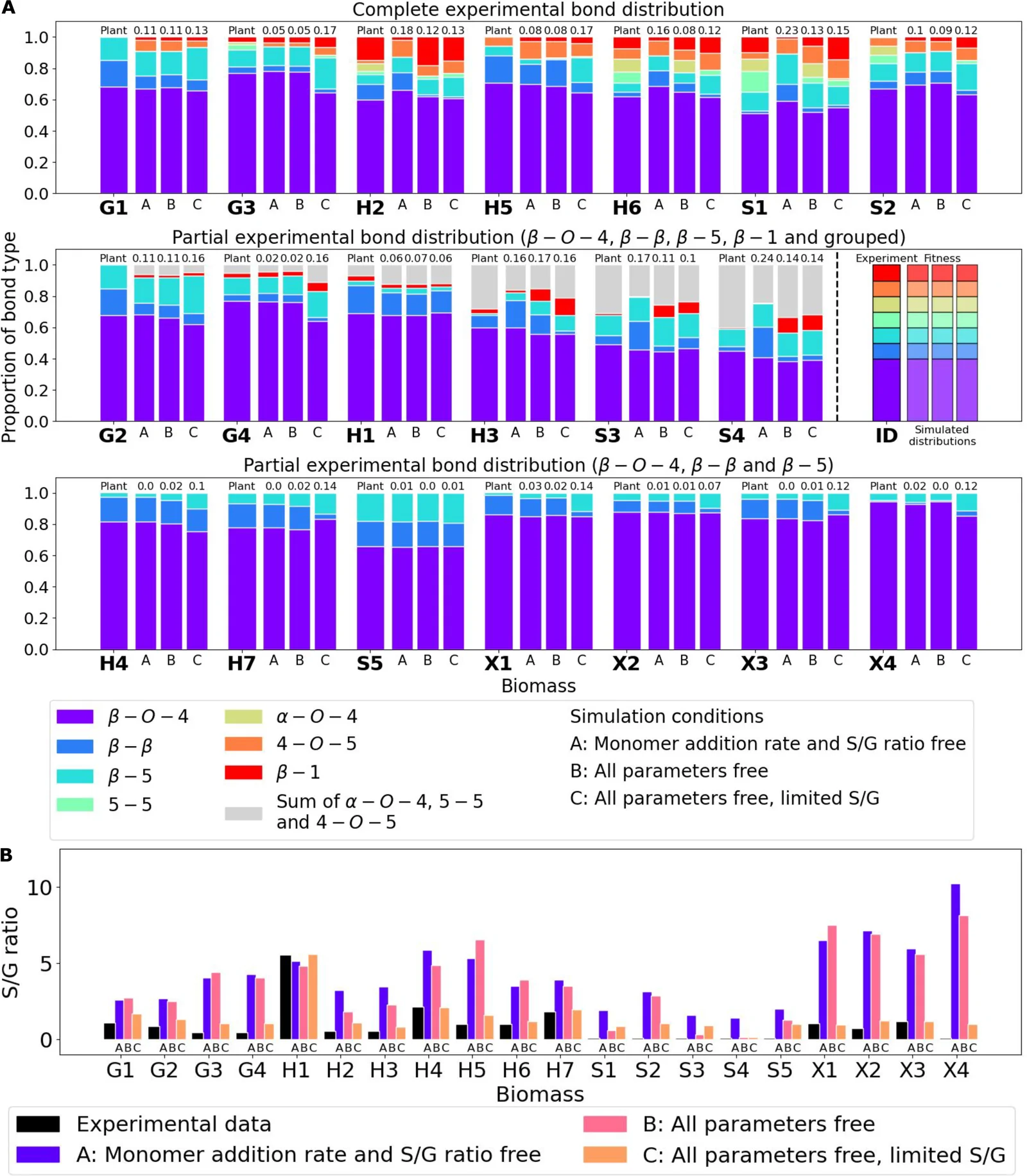
(i) Experimental and best-fit bond distributions for the 20 different biomasses listed in Table 1. Set A: The parameter monomer addition rate and S/G ratio are free to vary. Set B: All the parameters are free to vary. Set C: All the parameters are free to vary, except the S/G ratio, which can only vary in the range ±0.5 around the experimentally measured value (or in the range [0,1], if the experimentally measured value is less than 0.5). (ii) Experimental and simulated (but not fitted) S/G ratios for sets A (purple), B (pink), and C (orange).

The fitting procedure is illustrated in the middle box of Fig. 1. Because of the challenges encountered to accurately fit experimental bond distributions (see Fig. 3), in addition to the 4 input parameters of the Lignin-KMC algorithm (i.e., the S/G ratio, the minimum and maximum number of monomers, and the monomer addition rate), we also introduce 3 parameters that vary the free-energy barriers for the formation of the S–S, G–G, and S–G bonds. Since the minimum and maximum number of monomers are only meant to define the size of our simulation system, we systematically fix them at 10 and 500, respectively, for comparability of results across simulations, and to ensure that enough structures are generated for satisfying statistics. All other parameters can be selected for being optimised during the Fitting procedure (Fig. 1, middle box).

The Fitting procedure follows the same main steps as in our previously published algorithm for the lignocellulose degradation software PREDIG [24–27]. The algorithm uses recursive generations, where each of them contains subsets of parameter values randomised within a specified range, and simulates the lignin molecules’ growth using Lignin-KMC, with 50 repeats for each subset, to account for the stochastic essence of the simulation approach. The Euclidean distance between the average simulated and target experimental bond distribution is measured, and the subset with the lowest distance is selected. If this distance is lower than the one of the previous generation, a gradient descent approach is followed such that a new position in the parameter space is chosen along that gradient and the subsets of the new generation are randomly taken around it. Else, the subset with the lowest Euclidean distance at the previous generation is used again as a starting point such that the subsets of the new generation are randomly taken around it. This combination of random and directed searches progressively approaches a local or global optimum.

#### Structure library generation

The structure library generation is illustrated in the right box of Fig. 1. After the fitting procedure is finished (middle box of Fig. 1), the library of structures is generated (right box of Fig. 1) through a final simulation with the parameter values that produced the smallest Euclidean distance to the target experimental bond distribution during the upstream fitting procedure. The number of repeats is then increased from 50 to 200 to improve the statistics and account for the stochastic essence of the simulations. This typically yields a library of several thousands of lignin molecules ranging in size from DP 1 to 500.

## Results and Discussion

### Comparison of simulated versus experimental bond distributions and S/G ratios

Figure 3 shows the comparison between experimental data and simulation results for the bond distribution (panel i) and S/G ratio (panel ii) of the 20 different biomasses collected in Table 1 (see the Experimental data collection section). The 3 fitting conditions (sets A, B, and C, explained earlier in the Initial conditions identification section) vary in the number and the type of parameters selected for optimisation. The Euclidean distance shown above the bars (in panel i) quantifies the quality of the fit to the target experimental data, with a lower value indicating a better fit. Expectedly, the fits are best for the least constrained cases of the partial bond distributions (bottom row). In other words, if the bond distribution only accounts for the most abundant bond types, i.e., *β*–*O*–4, *β*–*β*, and *β*–5, the fitting algorithm is less constrained since it has a smaller output dataset to match, as compared to when the full bond distribution is to be reproduced.

For set A, the main bonds (*β*–*O*–4, *β*–*β*, and *β*–5) are well mimicked, while the less common 4–*O*–5 are consistently overexpressed. Additionally, in Fig. 3ii (set A), we consistently observe a strong overestimation of the S/G ratio across biomasses (except for H1). Hence, it is clear that simply varying the monomer addition rate is insufficient to accurately mimic the bond distributions while faithfully reproducing the experimentally measured S/G ratio. In sets B and C, parameters are introduced for scaling the free-energy barriers for the formation of S–S, G–G, and S–G bonds. In set B, the fits for the bond distributions are greatly improved for some of the softwoods (i.e., S1, S3, and S4), which were the worst ones in set A. In comparison to set A, in set B, either bond distributions are unchanged or the abundances of the less prevalent bonds (5–5, α–*O*–4, 4–*O*–5, and *β*–1) are significantly increased. In Fig. 3ii (set B), for H2, H3, H4, S1, S3, S4, S5, X3, and X4, the S/G ratio significantly decreases as compared to set A. However, in set B, even for the biomasses with a good fit in bond distribution (i.e., G3, G4, H4, H5, H6, H7, S5, X1, X2, X3, and X4), their S/G ratio still lies far away from the experimental value. To counter this limitation, in set C, we restrict the S/G ratio to vary only in the range ±0.5 around the experimentally measured value (or in the range [0,1], if the experimentally measured value is less than 0.5). Thereby, the S/G ratio overestimation is attenuated, while the fit to the experimental bond distribution is still acceptable.

Overall, only set C enables obtaining decent fits of the bond distributions, as well as an improved estimation of the S/G ratios compared to the experimentally measured values. It corresponds to a system where the energy barrier of S–S bond formation is higher than that of G–G, which must be lowered for better fits, alike the monomer addition rates. Together, this reflects a highly controlled process that ensures that monolignols rapidly react after they enter the system, thereby strongly reducing fluctuations (see Fig. B.1).

**Fig. 4.**
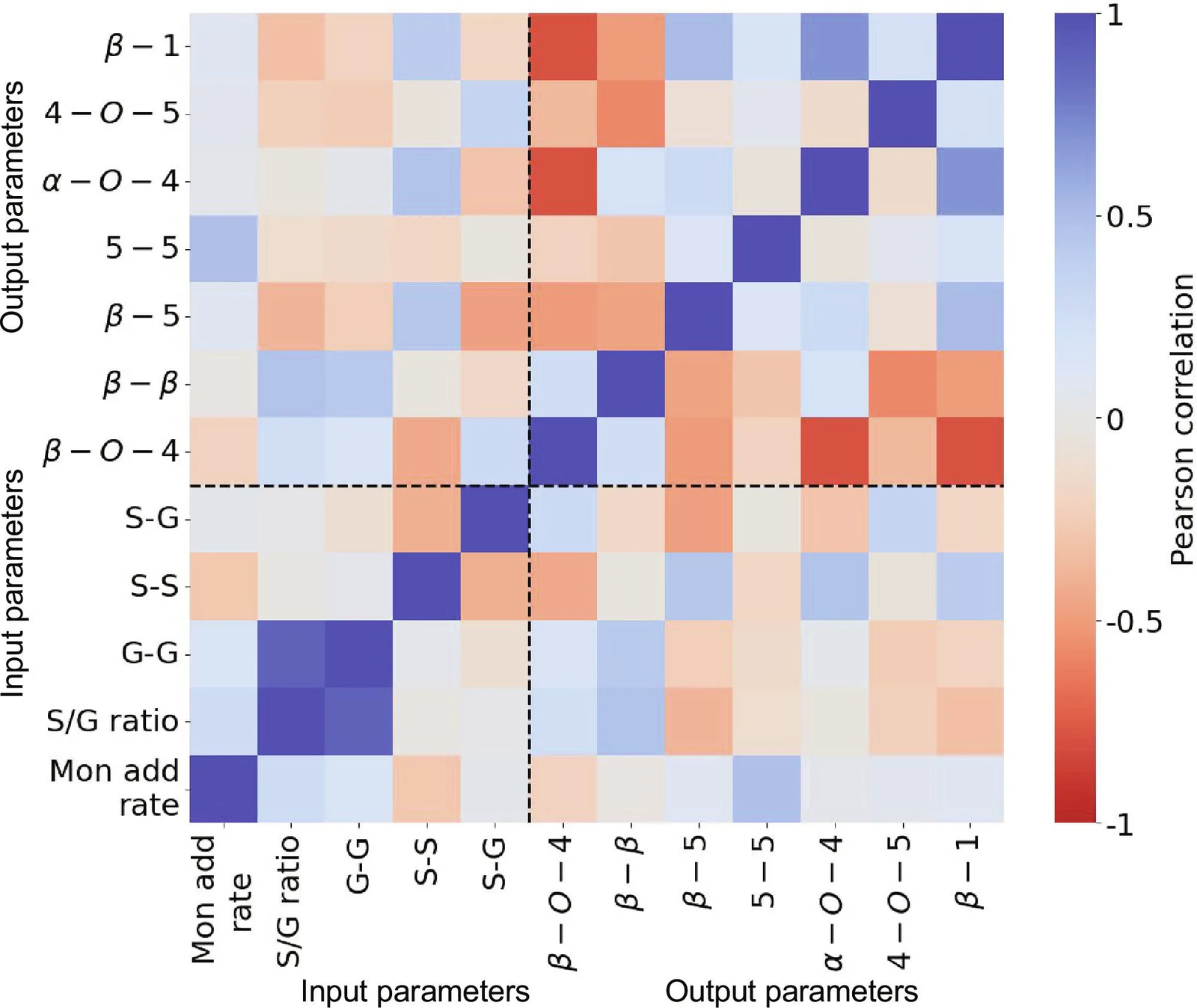
Matrix of Pearson correlation coefficients between input and output parameters from set C. Input parameters are monomer addition rate, S/G ratio, and the scaling parameters for the free-energy barriers for the formation of the S–S, G–G, and S–G bonds. Output parameters are the best-fitted bond distributions simulated by LigninFit for the 20 biomasses selected.

Using the best performing fitting procedure (set C), to establish relationships between the biosynthesis parameters and the resulting lignin molecules, in Fig. 4, we display the Pearson correlation coefficients between these parameters for all of our 20 best fits. The S/G ratio is strongly correlated to the scaling parameter for the G–G free-energy barriers, supporting our observation that the G–G energy barrier is key in tuning the S/G ratio. Additionally, strong anti-correlations are observed between bond abundances of *β*–*O*–4, and both α–*O*–4 and *β*–1. Slightly less pronounced is the anti-correlation between bond abundances of *β*–*β* and 4–*O*–5, and the correlation between bond abundances of α–*O*–4 and *β*–1.

From its chemical properties, it is known that a *β*–5 bond can only form if at least one G monolignol is involved in the reaction [28]. This is somewhat reflected in the weak anti-correlation between the *β*–5 bond abundance and the scaling factor for the energy barriers involving G monolignols, which ease bond formation as they reduce. Consistently, there is a correlation between the *β*–5 bond abundance and the scaling parameter for the S–S free-energy barrier, which, as it increases, indirectly favours the formation of S–G and G–G bonds, possibly involving *β*–5 linkages. Overall, the correlations between bond types and any of the scaling parameters for the free-energy barriers are not particularly strong, as the formation of the different bond types is dependent not only on monolignol interactions but also on their respective abundances, the structure and spatial orientation of the growing lignin molecules, and the monomer addition rate.

### Prediction of complete bond distributions

**Fig. 5.**
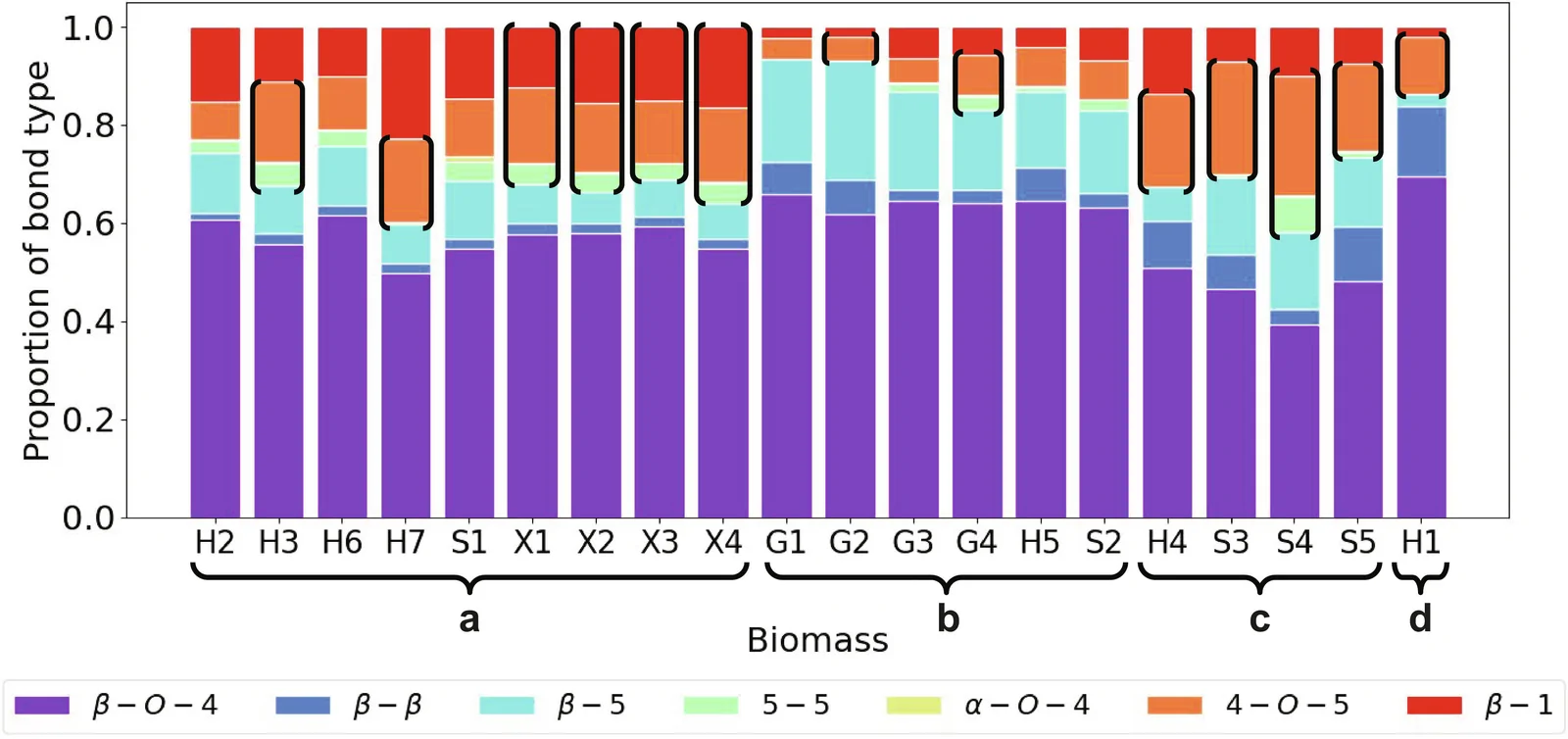
Bond distributions generated by LigninFit for each of the 20 best fits in set C. The biomasses are clustered (a, b, c, and d) based on the similarity of their average bond distribution. The round brackets indicate the specific parts of the bond distributions that are predicted by LigninFit but have not been experimentally measured.

Figure 5 shows the complete bond distributions generated for the best fits of the 20 biomasses in set C, including for those which have only partial bond distributions experimentally determined. For those biomasses with partial bond distributions, we predict the missing bond fractions with LigninFit; thereby, we also account for the less common linkage types: 4*–O–*5, *β*–1, 5–5, and α–*O*–4. Our simulation results highlight variations among biomasses, for instance, while the *β*–*O*–4 bond type is always the predominant one; it nonetheless varies between 40% (in S4) and 70% (in H1). Thus, we also grouped the bond distributions generated by LigninFit into 4 *k*-means clusters by minimising intra-cluster variances. Overall, although the clustering of the biomasses is mixed and does not fully obey the classification by the biomass source type (i.e., Gi, Hi, Si, and Xi), some clear patterns can be observed. All the other biomasses (Xi) are grouped in the largest cluster, *a*, along with most of the hardwoods (i.e., H2, H3, H6, and H7) and softwood S1. Bond distributions in cluster *a* are characterised by a high abundance of *β*–1 bonds. All the grasses (Gi) are sorted in the second largest cluster, *b*, which also contains H5 and S2. Bond distributions in cluster *b* are characterised by a higher abundance of *β*–5 bonds. Cluster *c* contains most of the softwoods (i.e., S3, S4, and S5), as well as one hardwood (H4). Bond distributions in cluster *c* are characterised by a higher abundance of 4–O–5 bonds. Eventually, hardwood H1 cannot be grouped with any of the other biomasses and is the sole occupant of the cluster *d*. It is characterised by a very high *β*–*β* and a very low *β*–5 bond abundance, consistently with its uniquely high S/G ratio, as observed in Fig. 3ii. It is noteworthy that even the same biomass, if reported by different experimental sources, might not lie in the same cluster (e.g., beech: H2 and H3 lie in cluster *a*, while H4 lies in cluster *c*; birch: H5 lies in cluster *b*, while H6 lies in cluster *a*; spruce: S1 lies in cluster *a*, while S2 lies in cluster *b*). These observations allow mitigating the widespread idea that the bond distribution is a strong indicator of the biomass type [29,30].

For each of the 20 best fits in set C, Table 2 shows the mean abundance for each bond type, for each biomass type (panel i), as well as the differences among biomass types for each bond type (panel ii). Across different biomass types, the softwoods (Si) are those showing the least abundance in *β*–*O*–4, although it remains their most abundant bond type. This is consistent with the experimental observation that *β*–*O*–4 abundance in softwoods is much less than in hardwoods [31].

**Table 2.** Characteristics of the bond distributions generated by LigninFit for each of the 20 best fits in set C. (i) Fraction of each bond type (columns) for each biomass type (rows). (ii) For each bond type (columns), *p* values for a Tukey HSD test to determine differences in bond abundances for pairs of biomass types (rows and sub-columns). Statistically significant results (*p* < 0.05) are highlighted with a “*”.

(i)	Bond type																											
Biomass type	β–O–4				β–β				β–5				5–5				α–O–4				4–O–5				β–1			
Gi	0.6404 ± 0.0171				0.0472 ± 0.0248				0.2039 ± 0.0327				0.0111 ± 0.0136				0.0009 ± 0.0014				0.0560 ± 0.0173				0.0406 ± 0.0227			
Hi	0.5894 ± 0.0725				0.0552 ± 0.0496				0.0957 ± 0.0422				0.0160 ± 0.0179				0.0013 ± 0.0029				0.1298 ± 0.0619				0.1127 ± 0.0700			
Si	0.5038 ± 0.0902				0.0523 ± 0.0379				0.1488 ± 0.0197				0.0298 ± 0.0276				0.0036 ± 0.0046				0.1701 ± 0.0697				0.0917 ± 0.0330			
Xi	0.5745 ± 0.0185				0.0205 ± 0.0012				0.0732 ± 0.0071				0.0391 ± 0.0048				0.0009 ± 0.0019				0.1440 ± 0.0141				0.1478 ± 0.0177			

(ii)	Bond type																											
	β–O–4				β–β				β–5				5–5				α–O–4				4–O–5				β–1			
Biomass type	Gi	Hi	Si	Xi	Gi	Hi	Si	Xi	Gi	Hi	Si	Xi	Gi	Hi	Si	Xi	Gi	Hi	Si	Xi	Gi	Hi	Si	Xi	Gi	Hi	Si	Xi
Gi	X	0.59	0.03*	0.49	X	0.99	1.00	0.75	X	0.00*	0.08	0.00*	X	0.97	0.45	0.17	X	0.99	0.28	1.00	X	0.84	0.01*	0.06	X	0.11	0.40	0.03*
Hi	0.59	X	0.14	0.98	0.99	X	1.00	0.47	0.00*	X	0.04*	0.66	0.97	X	0.58	0.22	0.99	X	0.29	0.99	0.84	X	0.45	0.96	0.11	X	0.87	0.64
Si	0.03*	0.14	X	0.38	1.00	1	X	0.59	0.08	0.04*	X	0.01*	0.45	0.58	X	0.87	0.28	0.29	X	0.29	0.01*	0.45	X	0.83	0.40	0.87	X	0.32
Xi	0.49	0.98	0.38	X	0.75	0.47	0.59	X	0.00*	0.66	0.01*	X	0.17	0.22	0.87	X	1.00	0.99	0.29	X	0.06	0.96	0.83	X	0.03*	0.64	0.32	X

Also, grasses (Gi) have both very low 4–*O*–5 and very high *β*–5 bond abundances. In panel ii, we observe that the abundance of *β*–*O*–4 bonds in softwoods (Si), where it is reduced, is only significantly different from that of the grasses (Gi). This is compensated by an opposite trend in the abundance of 4–*O*–5 bonds, for which softwoods (Si) show a significantly increased abundance, unlike grasses (Gi). Grasses (Gi) appear to also be significantly reduced in β−1 bonds as compared to the Other biomasses (Xi). Panel ii also highlights that the abundances of β–β, 5–5, and α–*O*–4 bonds are not significantly different across biomass types. Eventually, for *β*–5 bonds, only softwoods (Si) are not significantly different from grasses (Gi), and hardwoods (Hi) from other biomasses (Xi). These results suggest that *β*–5 bonds, which are the second or third most abundant ones in all biomass and involve either G-S or G–G bonds, represent a meaningful structural discriminant between biomass types [32,33].

### Molecular level analysis of bond distributions

**Fig. 6.**
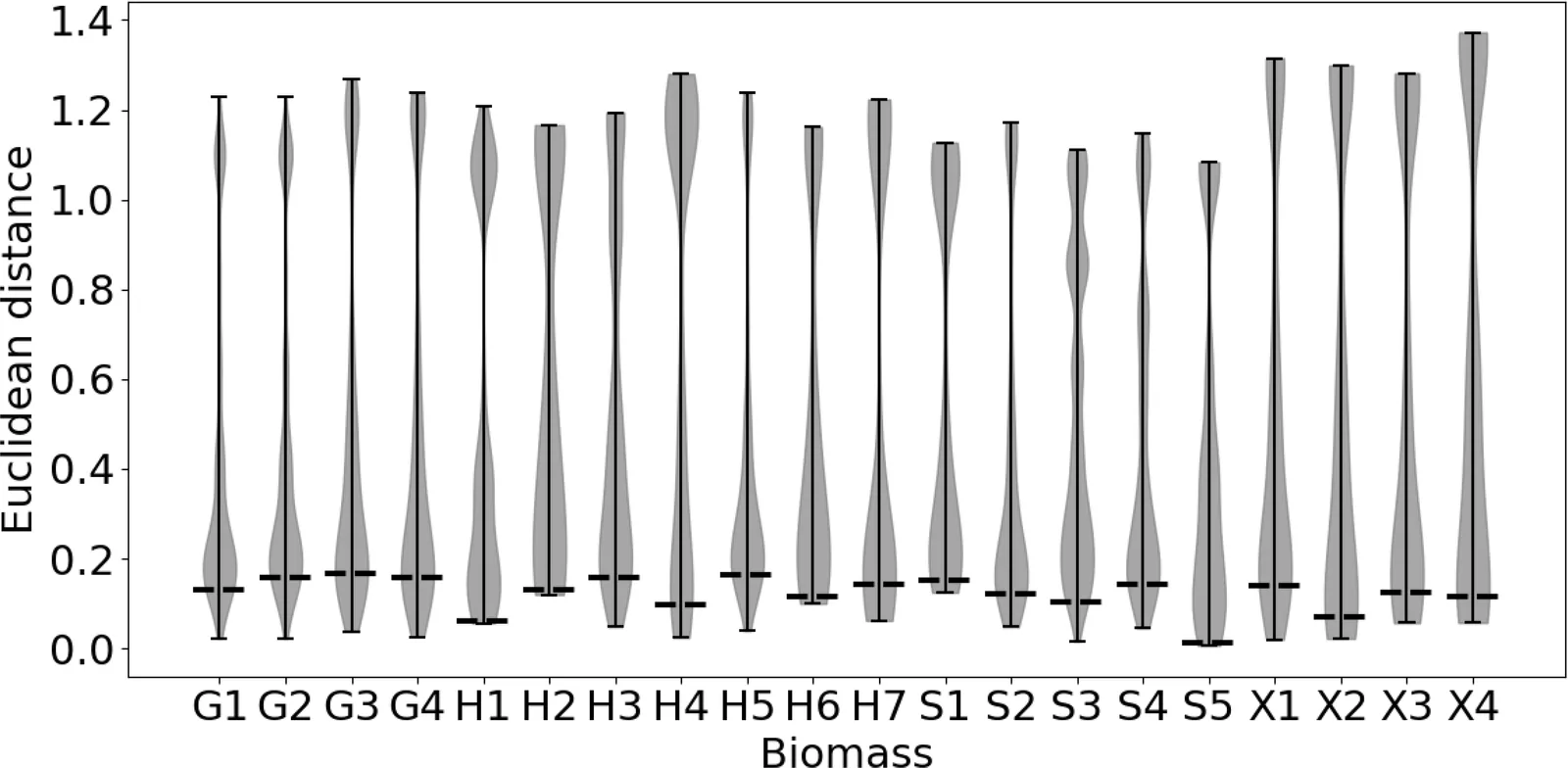
Violin plots showing distributions of the Euclidean distances between LigninFit simulated and experimentally determined bond distributions, measured at the single-molecule level, for lignin structure libraries generated for the best fits of each of the 20 biomasses in set C. The black lines show the range and the mean of each dataset.

Figure 6 shows the distribution of Euclidean distances between LigninFit simulated and experimentally determined bond distributions for the best fits of each of the 20 biomasses in set C. In these violin plots, the horizontal top and bottom bars denote the upper and lower limits of the distribution, respectively, while the Euclidean distance of the mean (i.e., the bond distribution displayed in Fig. 3i) is represented by the thick dashed line. For a majority of the biomasses, the distribution of Euclidean distances is clustered around the mean, while there exist a few cases in which the bimodal distribution shows a predominant second mode, with high Euclidean distances, like for biomasses H1, H2, H4, H7, S1, S3, X2, X3, and X4. This most likely arises from the large number of small linear molecules generated by LigninFit.

**Fig. 7.**
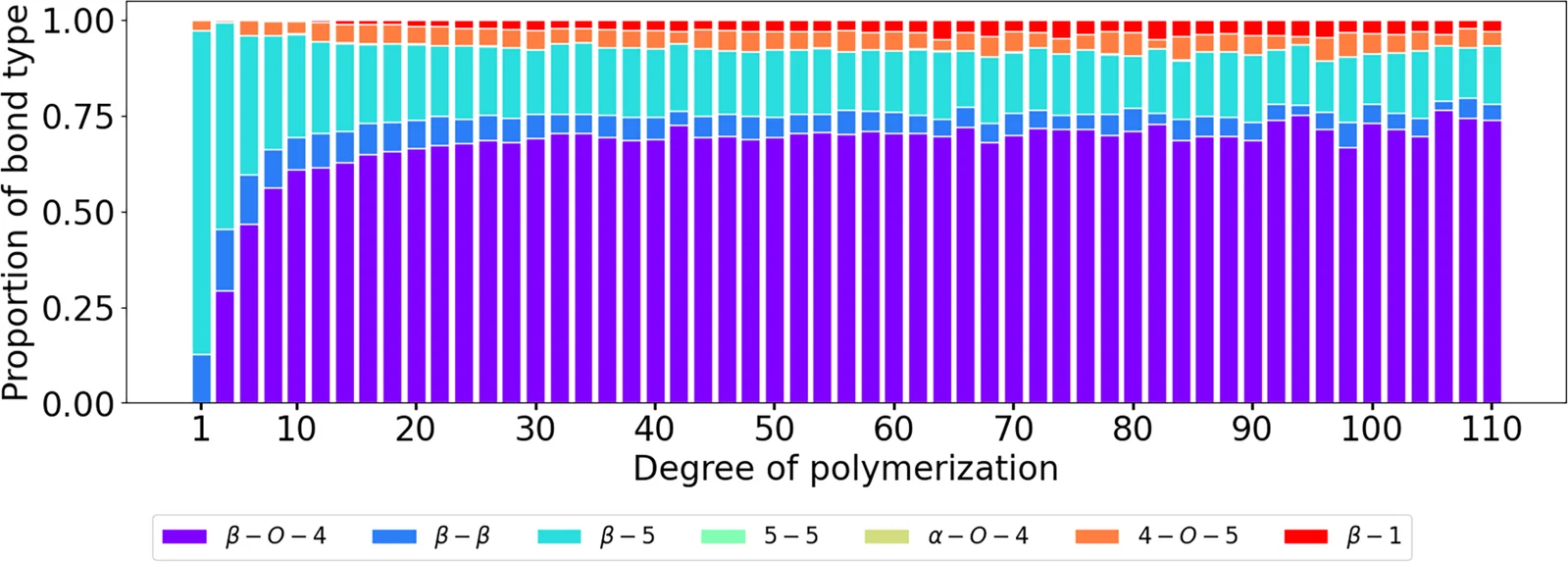
Bond distribution versus degree of polymerisation for the best fit of the illustrative biomass sample G1 in set C.

Figure 7 shows the variation of the bond distribution with increasing DP, for the best fit of the demonstrative sample G1 in set C. The bond distribution varies strongly going from DP1 to ca. DP30, beyond which it stabilises. As the DP increases, the abundances of *β*–5 and *β*–*β* bonds decrease, while those of *β*–*O*–4 and the rarer bonds 4–*O*–5 and *β*–1 increase. Overall, the proportion of bond types for small molecules (i.e., DP1 to DP10) is drastically different from that of larger molecules (i.e., DP ≥ 30) such that while the abundance of *β*–5 bonds strongly dominates for small molecules, it is only the second most abundant bond for larger molecules, far behind the *β*–*O*–4 bond. The contribution from all the other bond types remains marginal throughout the variation of DP, with a total contribution of ca. 10%.

### Branching properties

Branching is a key feature of lignin that shapes its properties and interactions in the cell wall [3]. Since the size and structure of lignin molecules are highly polydisperse, lignin branching is quite difficult to directly measure experimentally and is instead inferred from data. NMR spectroscopy, especially HSQC (heteronuclear single quantum coherence) and HMBC (heteronuclear multiple bond correlation), provides linkage information [29], while MALDI-TOF MS (matrix-assisted laser desorption/ionisation–time of flight mass spectrometry) offers complementary data on molecular weight distributions and fragments [34]. From these measurements, $M_{w}$, $M_{n}$, and the resulting polydispersity index (PDI) and branching factor (α) help assess branching patterns [35].

**Fig. 8.**
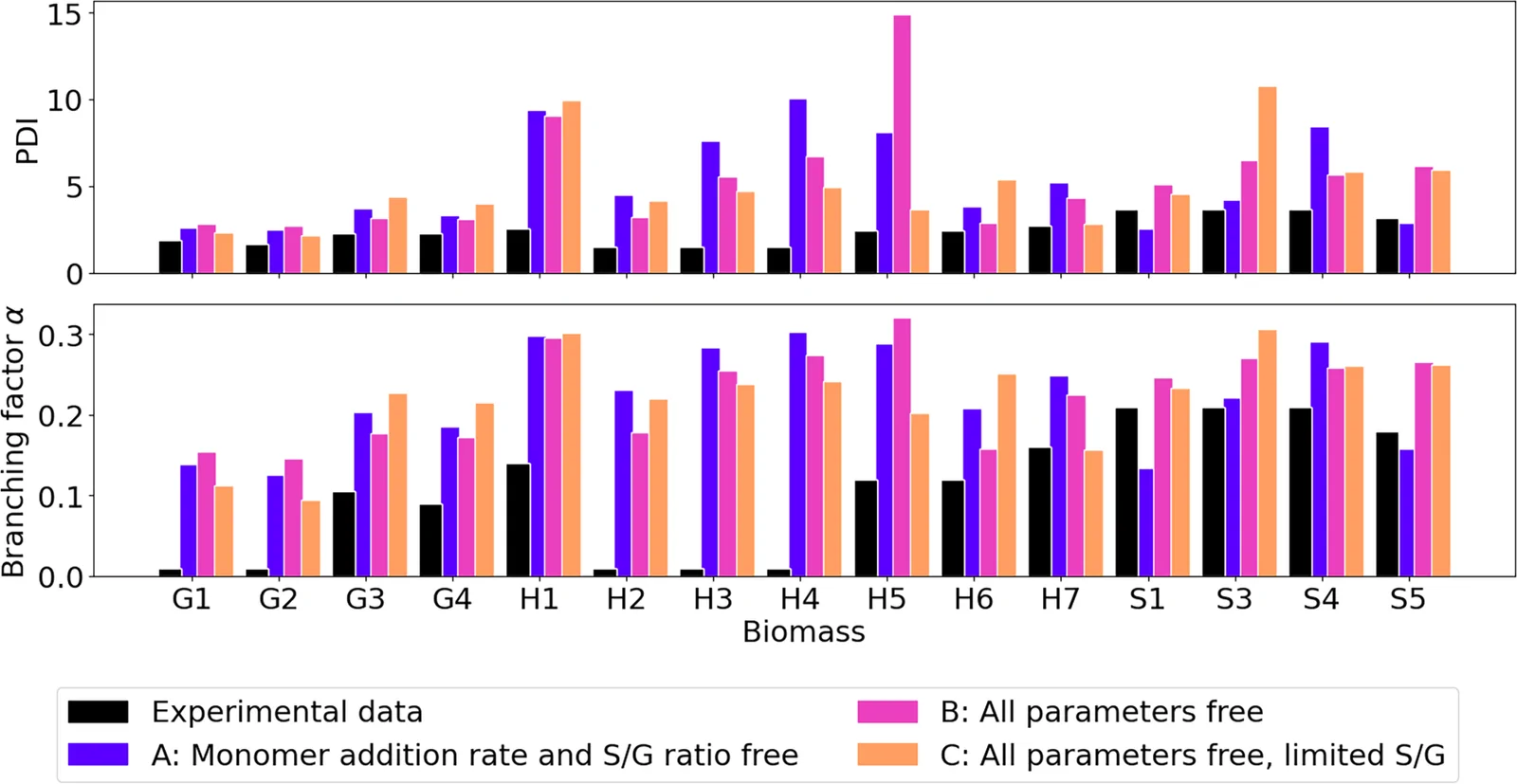
Measured at the ensemble level, for best fits from sets A, B, and C, following the same colour-code like in Fig. 3. (Top panel) Experimental and model predicted values for polydispersity index (PDI). (Bottom panel) Branching factor α. Only the 15 biomasses, for which the experimental data for PDI and α are available, are plotted here.

For the 3 fitting conditions (sets A, B, and C, explained earlier in the Initial conditions identification section), Fig. 8 shows the experimentally measured and the model-predicted PDI (top panel) and the branching factor α (bottom panel). The definitions of the PDI and the branching factor α are provided in Appendix C.1. Figure 8 (top panel) shows that for the grasses (Gi), the experimentally measured values of PDI are well matched with the LigninFit predictions for all the fitting conditions (i.e., sets A, B, and C). Apart from this, a good match with experimental values is observed in set A for H6, S1, S3, and S5, set B for H6, and set C for H5 and H7. Figure 8 (bottom panel) shows that the branching factor α is grossly overestimated for all biomasses except for S3 and S5 in set A, and H7 in set C. Overall, it clearly appears that the model much better predicts the PDI than the branching factor α. However, it must be noted that when comparing the top and bottom panels in Fig. 8, the experimentally measured branching factor α equals 0 for biomasses G1, G2, H2, H3, and H4, since their experimentally measured $\mathrm{PDI}<\frac{1}{0.63}$ [see Eq. (C.2)], which directly contributes to the severe overestimation of α by the model.

**Fig. 9.**
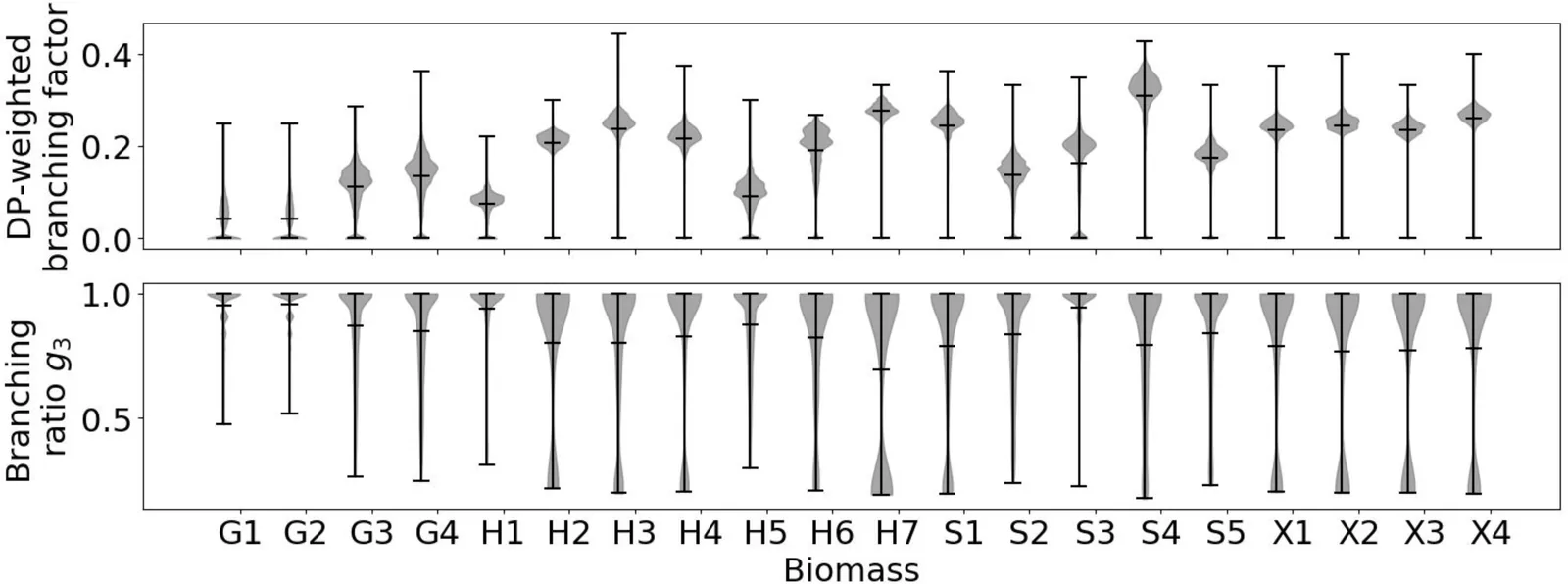
Violin plots showing distributions of properties, measured at the single-molecule level, for lignin structure libraries generated for the best fits of each of the 20 biomasses in set C. (Top panel) DP-weighted branching factor. (Bottom panel) Branching ratio $g_{3}$. The black lines show the range and the mean of each dataset.

Figure 9 shows the DP-weighted branching factor and the branching ratio $g_{3}$ for the lignin structure libraries generated for the best fits of each of the 20 biomasses in set C. For each biomass plot, the top and bottom bars show the range of the distribution, while the middle one represents the mean. The top panel shows that the DP-weighted branching factors for grasses G1 and G2 (both Miscanthus) are exceptionally low, consistent with results previously obtained for this type of biomass using the Pla and Yan metric of branching measurement [35]. The second lowest DP-weighted branching factors are for hardwoods H1 (eucalyptus) and H5 (birch), which should nonetheless be mitigated by the fact that the same biomass can show different results if reported by a distinct experimental source (e.g., for birch as H5 and H6). The bottom panel shows the branching ratio $g_{3}$ [see Eq. (C.3)], with a higher value indicating a linear molecule. The mode value of these plots is always at 1, which illustrates that our simulations generate a high abundance of small linear molecules. Biomasses G1 and G2 have the highest value of $g_{3}$, which points to the predominance of linear lignin molecules consistent with observations from the top panel. The second-highest values of $g_{3}$ are found for S3, H1, and H5, which only partially aligns with our findings in the top panel. Additionally, H5 showing a high value of $g_{3}$ counter-says its high PDI and branching factor α observed in Fig. 8. It is worth noting that DP-weighted branching factor measures branching density, not the total branching; hence, it cannot distinguish between large, highly branched macromolecules and small oligomers with few branches. Furthermore, while DP-weighted branching factor provides a convenient measure of branching density, it does not capture absolute molecular size, branching topology, or bond-type chemistry, and therefore cannot fully describe lignin architectural complexity on its own. Together, these highlight the versatility of the results depending on the branching metrics considered and, thus, the importance of studying systems as complex as lignin molecules with various quantitative measures.

Table 3 shows *P* values from the pairwise Tukey HSD statistical test [36] to determine which biomass groups differ significantly from one another, with respect to the 3 branching metrics defined in Figs. 8 and 9, namely, the branching factor α, the DP-weighted branching factor, and the branching ratio $g_{3}$ (for detailed definitions, see Appendix C). Lignin structures in Gi and Si show significantly distinct values in branching factor α and DP-weighted branching factor. Additionally, Gi and Hi are very close to being significantly distinct in the branching factor α and the DP-weighted branching factor. These findings are consistent with results previously obtained for this type of biomass using the Pla and Yan metric of branching measurement to grass lignins being generally linear compared to softwoods [35], and that grasses have a very low 4–O–5 abundance (Table 2). In contrast, the branching ratio $g_{3}$ does not show statistically significant differences across biomass groups, while there exist biomasses with clearly unique $g_{3}$ values, i.e., G1 and G2 (see Fig. 9). This suggests that the branching factor α and the DP-weighted branching factor are better quantitative descriptors of lignin branching than the branching ratio $g_{3}$, to differentiate between biomass types.

**Table 3.** *p* values calculated with a Tukey HSD statistical test to compare grouped means of the 3 branching properties for pairs of biomass types, for the lignin structure libraries generated for the best fits of each of the 20 biomasses in set C. Statistically significant results (*p* < 0.05) are highlighted with a “*”. Close to significant results are highlighted with a “†”.

	Branching property		
Biomass type pairing	Branching factor α	DP-weighted branching factor	Branching ratio g_3_
Softwoods (Si) and hardwoods (Hi)	0.5571	0.8669	0.1537
Softwoods (Si) and grasses (Gi)	0.0241*	0.0465*	0.3336
Grasses (Gi) and hardwoods (Hi)	0.0968^†^	0.078^†^	0.8995

## Conclusion

To bridge the knowledge gap in lignin structure formation, we develop a fitting algorithm, LigninFit, encapsulating Lignin-KMC, to quantitatively reproduce main features of lignin molecules that have been experimentally determined, and provide a deep analysis of further molecular properties that are hardly accessible with current experimental setups. We surveyed experimental literature to collect and curate quantitative data for lignin bond distributions, S/G ratios, DPs, and molecular weights for 20 biomasses. In order to replicate *in silico* lignin bond distributions for these 20 biomasses, we used LigninFit to simulate their formation under 3 fitting conditions, denoted by sets A, B, and C, varying in the number of model parameters optimised.

In set A, the optimised model parameters are the monomer addition rate and the S/G ratio. Under this condition, the bond distribution was not accurately reproduced, while the S/G ratio was grossly overestimated. To improve this, in set B, we optimised not only the monomer addition rate and the S/G ratio but also the energy barriers for the formation of S–S, G–G, and S–G bonds. Then, although the experimentally measured bond distributions were much better reproduced, the corresponding S/G ratios were still overestimated for most of the biomasses. Hence, eventually, in set C, the S/G ratio of each biomass was allowed to vary within a limited range around the experimentally measured value, while other parameters were equally treated as in set B. This allowed us to reproduce the bond distributions of the biomasses almost as well as in set B, while also better capturing the S/G ratio. However, it is important to remark that distinct sets of parameter values can equally well reproduce experimental data, thereby constituting local minima. This is especially true when experimental data provide only partial bond distributions, i.e., for H4, H7, S5, X1, X2, X3, and X4, which only have 3 bond types experimentally measured, i.e., *β*–*O*–4, *β*–*β*, and *β*–5. Here, it is important to note that the scaling parameters for the formation of the S–S, S–G, and G–G bonds that have been introduced in sets B and C should be interpreted as effective phenomenological parameters that reflect the variations within *in planta* microenvironments for lignin synthesis, and experimental measurement protocols rather than purely being physical free-energy corrections for an isolated system. Thus, caution should be exercised against interpreting them as direct biochemical kinetic rate constants.

Our analysis found a strong correlation between the scaling parameter for the free-energy barrier of G–G bond formation and the S/G ratio, along with strong anti-correlations between the abundances of *β*–*O*–4 and both α–*O*–4 and *β*–1 bonds. In addition, a weaker correlation was found between the abundances of α–*O*–4 and *β*–1 bonds, together with a weaker anti-correlation between *β*–*β* and 4–*O*–5 bonds. Furthermore, due to the involvement of at least one G monolignol in the formation of *β*–5 bonds, the abundance of those shows a weak anti-correlation with the scaling parameter for the free-energy barrier for G–G and S–G bond formation, as well as a weak correlation with the scaling parameter for free-energy barrier for S–S bond formation. The relative weakness of these correlations can be explained by the fact that the formation of different bond types is not only dependent on the monolignol interactions but also on their respective abundances, the structure and spatial orientation of the growing lignin molecules, and the monomer addition rate. Our results also suggest that the concentration of monomers is not the main factor controlling the dynamics of formation of lignin molecules, but that one should also consider the rate at which the reactants are added to the simulation environment. Adding the reactants at a slow rate allows for tightly controlling the dynamics of the system, ultimately leading to lignin molecules with better defined structural and compositional properties.

LigninFit enables the prediction of the complete bond distribution for those samples that have only incompletely determined experimental ones. For instance, 4–*O*–5*, β*–1, 5–5, and α–*O*–4 bond types are challenging to measure experimentally due to their typically low abundance and overlapping peaks in NMR/HSQC measurements [37] such that predicting them as we do with LigninFit might be of particular relevance to the experimentalist community. When grouping the complete bond distributions into 4 *k*-means clusters, it turns out that those do not fully correspond to the classification by the biomass type, i.e., Gi, Hi, Si, and Xi. Interestingly, even a given biomass type, if reported by different experimental sources, might be grouped into different clusters. This mitigates the widespread idea that bond distributions are strong indicators of the biomass type.

Analysis of the branching parameters of the lignin libraries generated by LigninFit highlights significant differences between grass and softwood lignins, with the grass lignins being predominantly linear, consistent with results from previous studies using similar metrics [35]. Moreover, our study shows that among the 3 branching metrics employed, i.e., the branching factor α, the DP-weighted branching factor, and the branching ratio $g_{3}$, the former 2 allow better differentiating between biomass types than the latter does. Eventually, we observed that the relative bond abundances strongly vary with the DP for small molecules, while they stabilise beyond ca. DP30.

In the experimental literature, there subsists an extensive debate on the formation of lignin branches *in planta* [2,35,38,39]. This mainly results from the challenges of experimentally characterising these branches. The processes used to extract lignin are chemically harsh, often utilising extreme pH or temperature, which typically causes bond breakage and oxidation, leading to side-chain oxidation and further lignin polymerisation. Klason lignin [40], milled wood lignin (MWL), and Kraft lignin [41] differ significantly in their extraction methods, impacting their structural properties. Klason lignin [40], obtained via sulfuric acid hydrolysis, is used primarily for quantifying lignin content, which may be overestimated due to residual non-lignin materials. MWL, extracted through mild enzymatic or chemical methods, instead retains a more native lignin structure, providing detailed structural insights, yet with a smaller yield. Eventually, Kraft lignin, a byproduct of the Kraft pulping process, is structurally altered and sulfur-rich, making it suitable for industrial uses but less adapted for precise structural studies. Beyond their individual advantages and limitations, overall, these methods fail to accurately depict *in planta* lignin properties, which ultimately emphasise the need for modelling methods to help rationalise experimental data. Toward this aim, LigninFit not only enables quantitatively reproducing main features of lignin that have been experimentally determined but also highlights the key factors influencing lignin formation dynamics, such as the monomer addition rates and the energy barriers for the formation of G–G and S–G bonds. Furthermore, it reveals unexpected similarities and differences across biomass types, and identifies key parameters for discriminating among them, such as the relative abundances of *β*–*O*–4, *β*–5, 4–*O*–5, and *β*–1 bonds, as well as branching metrics (i.e., the branching factor α and the DP-weighted branching factor), thereby contributing to guide future experimental endeavours.

In particular, computer-assisted methods allow screening for parameter ranges and resulting lignin structures that can be difficult to isolate experimentally at sufficient yield, while testing their properties could advance biofuel production and bioplastics development [42]. For instance, understanding these atypical bonds with low abundances can lead to more efficient lignin depolymerisation processes, enhancing its downstream valorisation [43,44].

## Data Availability

The model code and data, together with the scripts for reproducing the figures, are provided in the GitLab repository accessible via https://github.com/LianneGahan/LigninFit.

## Appendix A. Glossary

H: p-coumaryl alcohol

G: Coniferyl alcohol

S: Sinapyl alcohol

DFT: Density functional theory

S/G: Ratio of S and G monolignols

MWL: Milled wood lignin

KMC: Kinetic Monte Carlo

NMR: Nuclear magnetic resonance

HSQC: Heteronuclear single quantum coherence spectroscopy

HMBC: Heteronuclear multiple bond correlation

MALDI-TOF MS: Matrix-assisted laser desorption/ionisation–time of flight mass spectrometry

DP: Degree of polymerisation

PDI: Polydispersity index

SMILES: Simplified molecular input line entry system

GC/MS: Gas Chromatography/Mass Spectrometry

## Appendix C. Branching definitions

### Appendix C.1 The polydispersity index (PDI) and branching factor α

The polydispersity index (*PDI*) of a molecular distribution can be calculated as the ratio

$$\mathrm{PDI}=\frac{M_{w}}{M_{n}},$$ (C.1)

where $M_{w}$ is the weight average molecular weight ($M_{w}=\frac{\sum M_{i}^{2}N_{i}}{\sum M_{i}N_{i}}$) and $M_{n}$ is the number average molecular weight ($M_{n}=\frac{\sum M_{i}N_{i}}{\sum N_{i}}$), with $N_{i}$ being the number of molecules that have the molecular weight $M_{i}$. In the unlikely case that all the polymer molecules have identical weights (a pure mono-disperse sample), the ratio $M_{w}/M_{n}=1$.

The branching factor α is a function of the polydispersity index (PDI) and can be expressed as follows:

$$\alpha =\left\{\begin{array}{ll} -0.58\cdot {\mathrm{PDI}}^{-1}+0.36 & \text{if}{\mathrm{PDI}}^{-1}<0.63\\ 0 & \text{if}{\mathrm{PDI}}^{-1}\geq 0.63 \end{array}\right.$$ (C.2)

Thus, a higher value of α indicates a more complex and branched structure.

### Appendix C.2 DP-weighted branching factor

For each molecule, the branching is defined as the ratio of $N_{B}/\mathrm{DP}$, where $N_{B}$ is the number of branches and *DP* is the total degree of polymerisation for that molecule. The mean branching factor is then weighted according to DP such that larger molecules contribute more in the average.

### Appendix C.3 Branching ratio $g_{3}$

The branching ratio $g_{3}$ is used to characterise branched polymers of heterogeneous weight. It characterises trifunctional branching and can be expressed as:

$$g_{3}={\left({\left(1+\frac{n_{b}}{7}\right)}^{\frac{1}{2}}+\frac{4n_{b}}{9\pi }\right)}^{-\frac{1}{2}},$$ (C.3)

where $b_{w}$ is the weight-average number of branch units per molecule and $n_{b}$ is the average number of branch units per molecule. A low value of $g_{3}$ indicates a highly branched structure.
